# Spiral Wrap-Around Technique in the Reverse Radial Artery Fasciocutaneous Forearm Flap for Thumb Reconstruction: A Report of An Innovative Technique

**DOI:** 10.7759/cureus.50999

**Published:** 2023-12-23

**Authors:** Othillah M Moazin, Tanveer A Bhat, Faryal Suraya, Rakan H Alelyani, Majd Assad, Hana A Alazzmi, Hussain Alobaidi, Sultan Alaqil, Emad A Alfadhel, Hussan Birkhez Shami, Anas Aljasir

**Affiliations:** 1 Department of Plastic and Reconstructive Surgery, King Saud Medical City, Riyadh, SAU

**Keywords:** thumb, radial forearm flap, osteoplastic reconstruction, replantation, digital amputation

## Abstract

For amputation of the thumb in any age group, microsurgical replantation is the gold standard over other osteoplastic thumb reconstruction methods as it restores the form, function, and cosmesis of the thumb better. In the osteoplastic reconstruction of the thumb, usually, a pedicled groin flap or a reverse radial artery forearm flap is used to provide the soft tissue cover, and each of these flaps has its own set of merits and demerits. The reverse radial artery forearm flap can be used as a fascial or fasciocutaneous flap in an islanded or peninsular form. Using it as a fasciocutaneous forearm flap creates a donor site secondary defect that needs skin grafting, leading to an unsightly permanent cosmetic deformity in the forearm. We report a case of a 25-year-old male patient who underwent post-traumatic near-total thumb amputation following a crush avulsion injury in whom revascularization failed, and we successfully performed osteoplastic thumb reconstruction using the same phalanges as skeletal support and the reverse radial forearm flap as soft tissue cover. We devised a novel but simple spiral wrap-around technique in the reverse pedicled fasciocutaneous flap by rearranging the dimensions, changing the length-to-width ratio to 5:1, and then wrapping this strip of flap spiraling around the bony skeleton with primary closure of the donor site.

## Introduction

As reported in the literature, the thumb constitutes around 40% of hand function, and to restore this much thumb function following traumatic thumb amputation, microsurgical replantation is the gold standard of care if feasible; otherwise, reconstruction is the other alternative [1,2]. With the advent of microsurgery, effective replantation rates for both complete and subtotal amputations range from 55% to 93% among institutions, and the most frequent complications following replantation are venous and arterial thrombosis [3,4]. In thumb amputations that are not replantable, different techniques such as composite free tissue transfer including toe-to-thumb transfer, reverse pedicled osteocutaneous radial forearm flap, pollicization, the first web space deepening using flaps or skin grafts, metacarpal lengthening by distraction osteogenesis, osteoplastic reconstruction, the first dorsal metacarpal artery island flap, and osseointegrated thumb prosthetic replacement have been used [1-7].

In the traditional osteoplastic reconstruction of the thumb, a corticocancellous bone graft harvested from the iliac crest serves as a skeletal support, which is then wrapped around by a pedicled groin flap and divided after three to four weeks in the second stage with primary closure of the donor site. A better alternative to a groin flap is a reverse radial artery forearm flap (RRAFF), which is readily obtainable in different dimensions, has a good color match, is pliable and thin, and can be raised as a composite flap depending on the requirements, besides being a single-stage procedure. However, one drawback associated with this flap is the creation of the secondary defect, which usually needs split-thickness skin grafting, ending up in a visible permanent cosmetic deformity over the forearm.

Here we present a case of right thumb amputation in a 25-year-old right-hand dominant patient in whom we performed successful single-stage thumb reconstruction by reusing the same amputated portion as a bone graft combined with a reverse radial forearm fasciocutaneous flap spirally wrapped around the thumb skeleton. We have incorporated a novel modification in the reverse radial forearm flap by rearranging the dimensions and raising a flap with a length-to-breadth ratio of 5:1, which enabled us to close the donor site, thereby primarily preventing the creation of permanent cosmetic deformity.

## Case presentation

**Figure 1:**
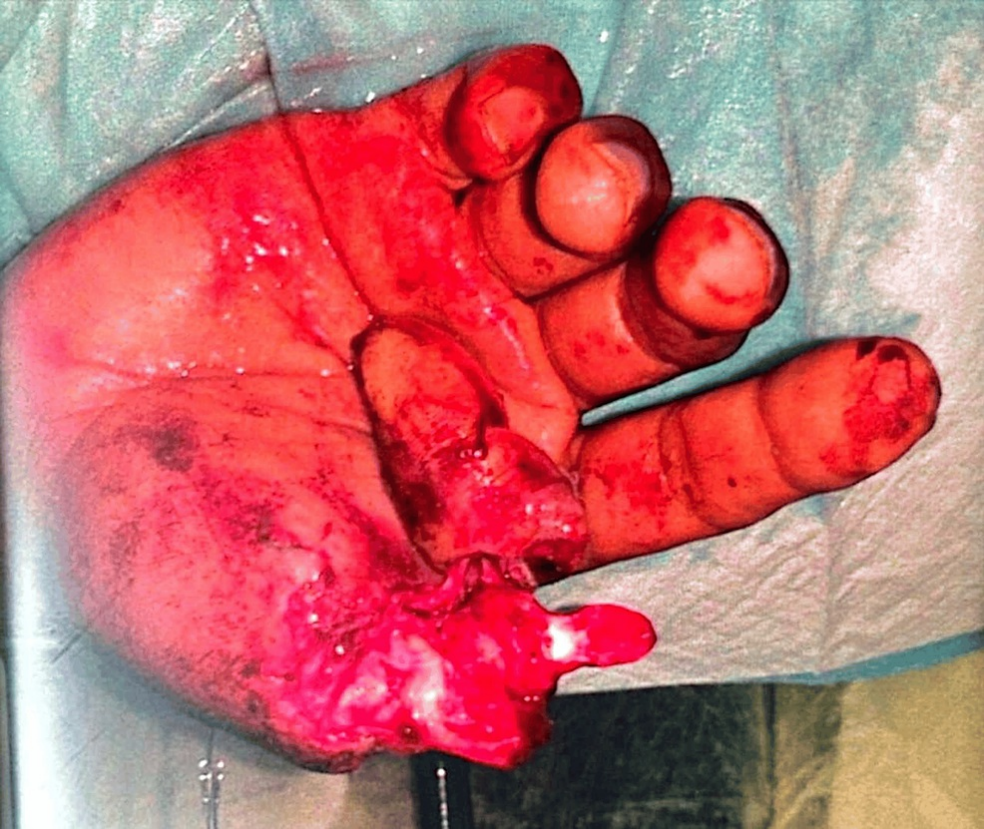
Preoperative picture showing near-total amputation of the right thumb with soft tissue avulsion of the residual stump.

A 25-year-old right-handed male, non-smoker, with no known comorbidity, was brought to the emergency department of our medical city with a right-hand near-total thumb amputation and an avulsion injury that resulted from a fall of a heavy gas cylinder on his right hand directly over the right thumb (Figure 1).

**Figure 2:**
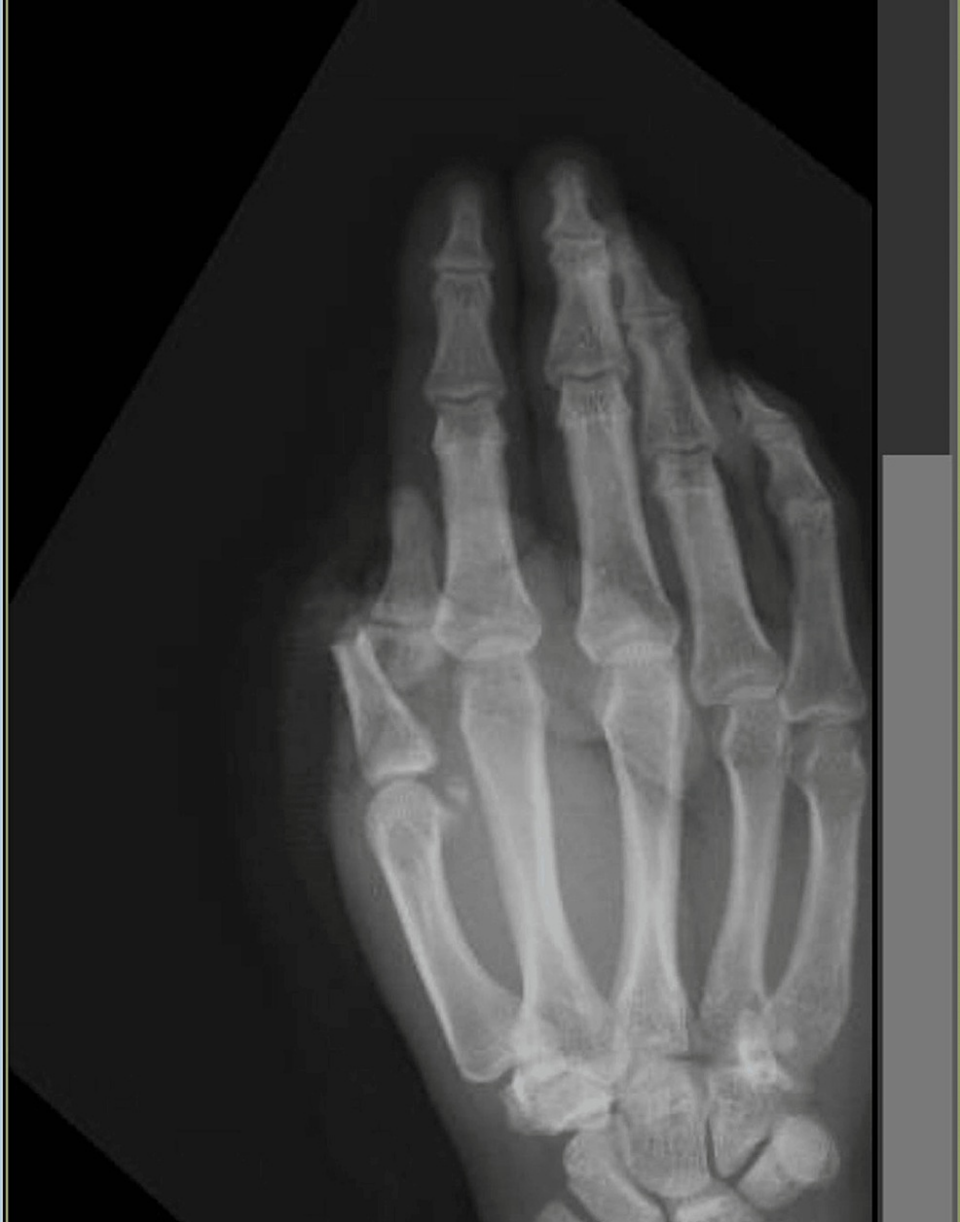
Posteroanterior view of the X-ray of the right hand showing displaced fracture through the neck of the proximal phalanx of the thumb.

The patient was assessed, and life-threatening injuries were ruled out by the emergency physician using the latest advanced trauma life support guidelines. The patient received tetanus prophylaxis, broad-spectrum antibiotics, and multimodal analgesia parenterally. Two-hand X-ray views, including a posteroanterior view and lateral views, were advised, along with a hemogram, blood chemistry coagulation profile, and blood grouping with cross-matching. The hand was examined under tourniquet control and revealed near-total amputation around the metacarpophalangeal joint, with the distal part hanging by only the flexor tendon and the contused neurovascular pedicles, with a significant avulsion injury of the proximal residual stump. The amputated part had compromised vascularity. The hand X-ray showed amputation through the shaft of the proximal phalanx (Figure 2).

**Figure 3:**
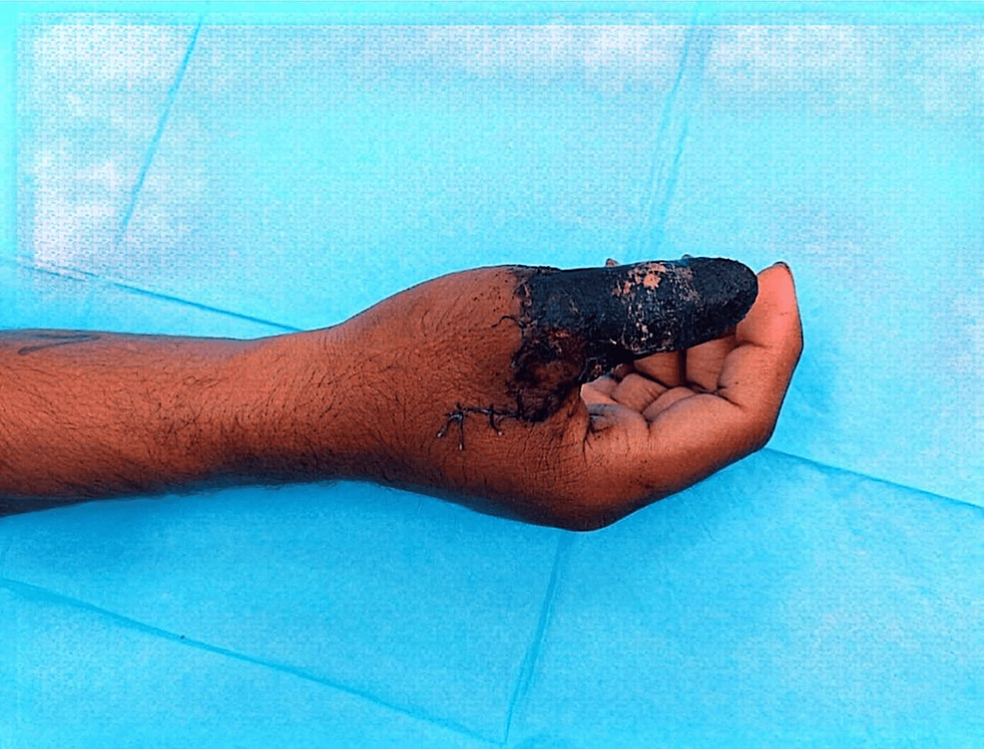
Picture showing dry gangrene of the right thumb post-revascularization performed for the avulsion injury.

The severity of the injury and the treatment options, along with the risks and benefits of each option, were explained to the patient, and the patient agreed to explore and possibly undergo revascularization of the thumb. An informed consent was documented, and the patient was taken to the operating room. Revascularization of the thumb was performed, and the patient was put on a post-revascularization treatment protocol including intravenous fluids, antibiotics, patient-controlled analgesia, heparin infusion, and 2 hourly monitoring for the vascularity of the thumb. The patient developed arterial insufficiency on the second post-operative day (POD), which was progressive and eventually ended in dry gangrene (Figure 3).

**Figure 4:**
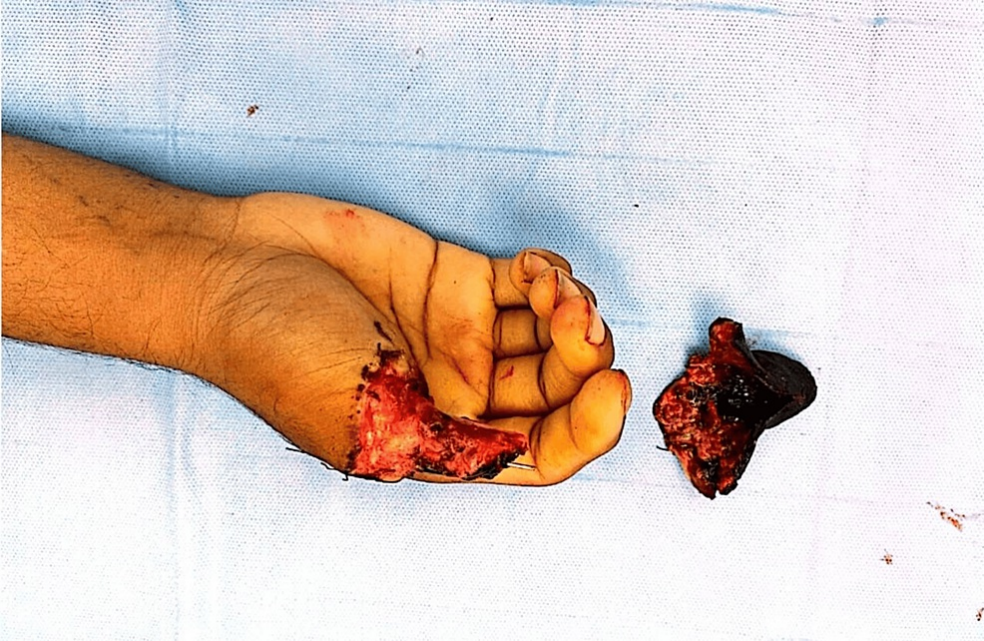
Intra-operative picture showing the residual stump of the right thumb after the debridement of the non-viable tissue.

After a thorough discussion with the patient, keeping in view his age, the dominance of the hand, and the level of amputation, we offered osteoplastic reconstruction of the thumb using a reverse radial forearm flap, which the patient agreed to, and the consent was penned down. The patient was subjected to general anesthesia with orotracheal intubation and operated under tourniquet control. After a proper surgical debridement and curettage, the wound was lavaged, and the resultant defect was assessed (Figure 4).

**Figure 5:**
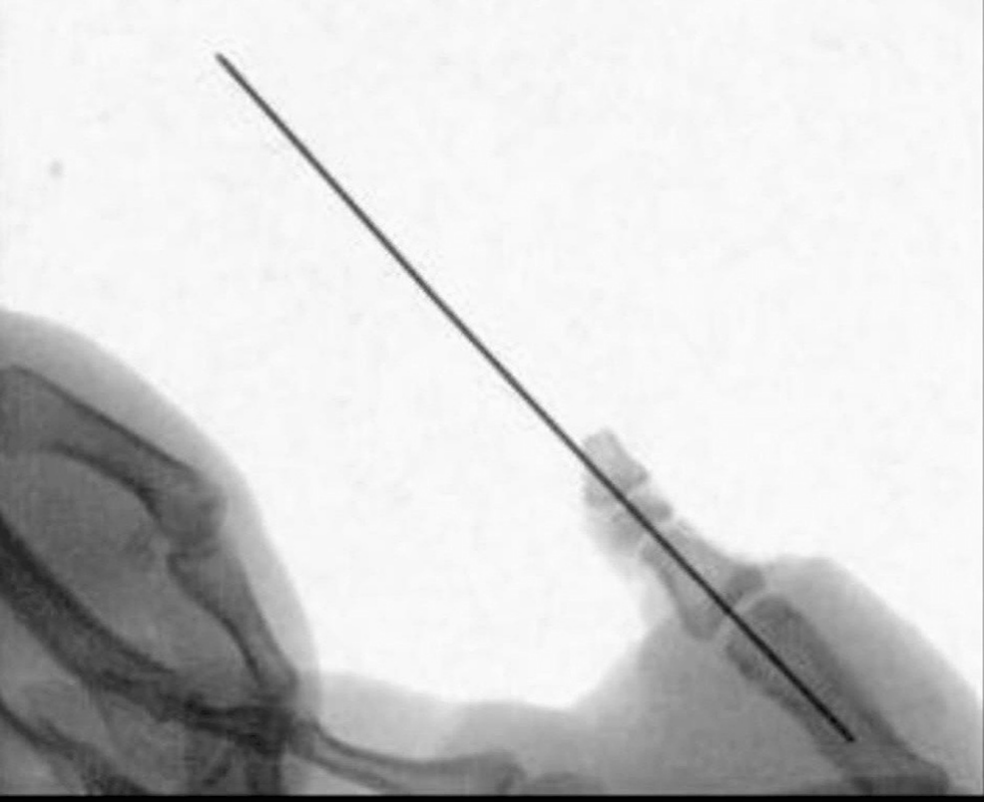
Picture showing fixation of the displaced fractured with a 1-mm-thick axial Kirschner wire.

A 1.0-mm Kirschner wire was used to fix the distal amputated bone fragment to the proximal stump axially under C-arm guidance (Figure 5).

**Figure 6:**
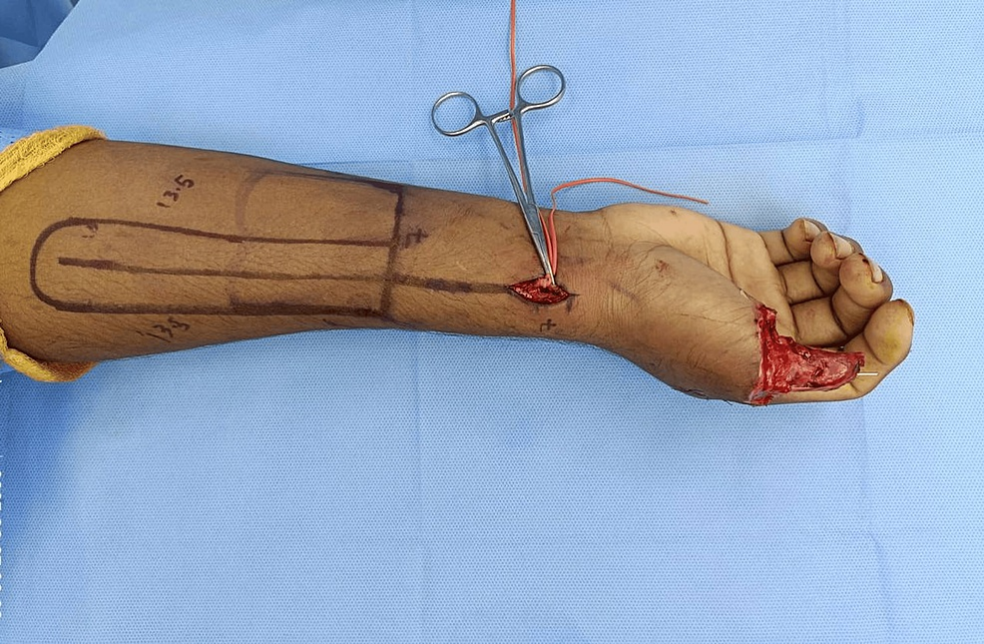
Intra-operative picture showing two flap designs: the traditional RRAFF and our newly designed flap (long one) for thumb reconstruction. RRAFF, reverse radial artery forearm flap

Intra-operatively, Allen’s test was repeated again to confirm palmar arch patency. Using a handheld Doppler, the radial artery was outlined with one-third of the flap width radial and two-thirds ulnar to it, and, finally, the pivot point was marked (Figure 6).

**Figure 7:**
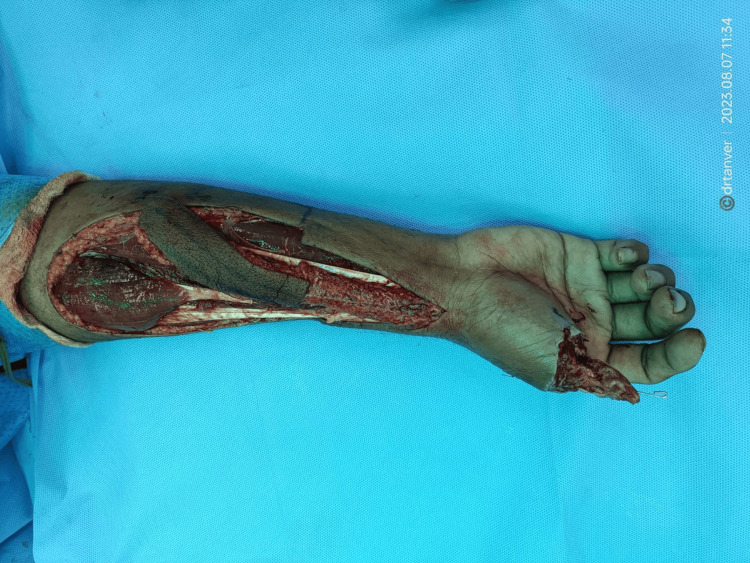
Intra-operative picture showing the harvested strip-like RRAFF. RRAFF, reverse radial artery forearm flap

Here we incorporated a new technique in the flap design by rearranging the dimensions to obviate the creation of the secondary defect and enable the primary closure of the donor site. We increased the length, decreased the width of the flap, and planned to harvest a long strip of the flap with the primary closure of the donor site. The islanded flap was raised with dissection, beginning from the ulnar to the radial border transversely and from the distal to the proximal end of the flap base vertically in the subfascial plane. We raised a 15-cm-long and 3-cm-wide flap with a pedicle length of 8 cm from the pivot point to the base of the flap in the subfascial plane with a length-to-breadth ratio of 5:1 (Figure 7).

**Figure 8:**
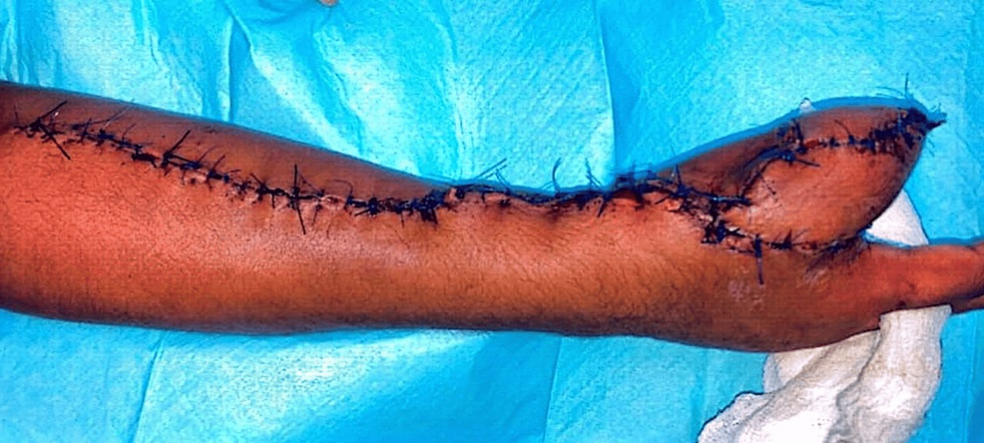
Picture showing primary closure of the donor site over the forearm by interrupted Proline 2-0 sutures.

This long strip-like flap was wrapped around the bony skeleton spirally, suturing the superior edge of the inferior curve with the inferior edge of the superior curve of the spiral, and the donor site was closed primarily using Proline 2-0 (Ethicon, Cornelia, GA, USA) (Figure 8).

**Figure 9:**
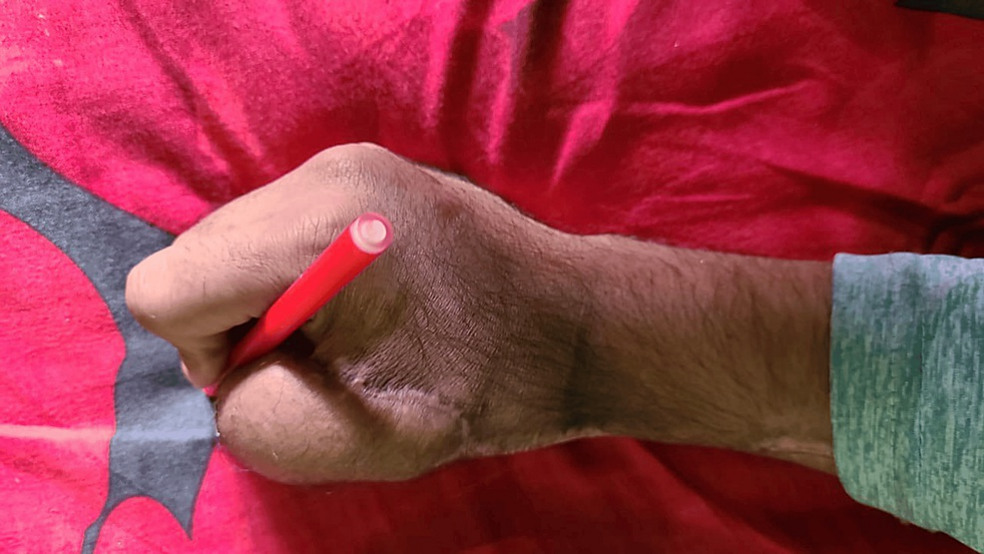
Follow-up picture after five months showing the reconstructed thumb with satisfactory pincer grasp.

The wounds were dressed, and a thumb spica slab was applied. Two hourly monitoring of the flap was done using clinical methods supplemented by handheld Doppler for the first 24 hours and then four hourly for the next four days. The first dressing was done on the fifth POD, and on the 10th POD, the patient was discharged with outpatient department follow-up every fortnightly for two months and then monthly for five months (Figure 9).

## Discussion

In traumatic amputation or near-total amputation, replantation or revascularization of the thumb is the treatment of choice, respectively, whenever feasible. However, when the amputated thumb is not replantable or when there is post-replant failure because of a severe avulsion injury, as seen in our patient, other methods of thumb reconstruction are considered depending on the level of amputation and patient acceptability.

One of these methods is the osteoplastic reconstruction of the thumb, which is well-known among hand surgeons. In this method, traditionally, a groin flap is used to wrap the new thumb skeleton, which is usually an iliac bone graft. The continuous three-week hand attachment to the groin, gravitational edema, delayed initiation of occupational therapy, and double-stage surgery are among the demerits associated with this flap [8]. Therefore, the reverse pedicled radial fasciocutaneous forearm flap is a better alternative to the groin flap; however, this flap is associated with sacrificing a major vessel to the hand and the resultant permanent cosmetic deformity of the forearm. However, the present research shows radial artery ligation does not significantly affect hand circulation [9]. Besides, we can reconstruct the radial artery defect using a venous graft or take a radial artery preserving perforator-based flap.

We incorporated a novel modification in this flap for the osteoplastic reconstruction of the thumb, which obviated the creation of the secondary defect, enabling us to close the donor site primarily and without any need for skin grafting and to avoid the formation of resultant permanent aesthetic deformity of the forearm. Although a hand surgeon has an armamentarium of flap choices for the reconstruction of the hand involving the thumb, reverse radial artery forearm flap (RRAFF) is commonly used as it is reliable and provides pliable tissue that drapes the bones well, gives better contour, has constant anatomy, and is robust [10,11].

The reconstructive hand surgeon has the flexibility to harvest different-sized flaps as demanded by the size of the defect because of the adequacy of the soft tissue of the flexor aspect of the forearm, and even the whole of this flexor aspect skin can be raised as RRAFF, leaving just a 2 cm cuff of skin ulnodorsally [12]. A traditional RRAFF has three main drawbacks when used for reconstruction of the thumb, including sacrificing the radial artery, donor site morbidity, and radial nerve neuroma formation [10]. Weinzweig et al. [12] and Jeng and Wei [13] reported having successfully raise the RRAFF on the radial artery perforators, thereby preserving the radial artery to perfuse the hand.

However, while searching the literature, we could not come across a single case in which the donor site was closed primarily after harvesting the RRAFF for thumb reconstruction, and our patient is probably the first such case in which we closed the donor site primarily without affecting the length of the neo-thumb by incorporating the spiral wrap-around technique. Our technique has the added advantage of reducing the formation of distressing radial nerve neuroma, as we harvested just a 3-cm-wide flap, which enabled us to minimize the dissection on the radial side of the flap, thereby decreasing the chances of radial nerve injury.

## Conclusions

Our new spiral wrap-around technique of RRAFF reconstruction of the thumb is a new and effective technique to avoid the creation of donor site soft tissue defect over the forearm and enables primary closure of the donor site.
